# Thermally Activated Delayed Fluorescence in Commercially Available Materials for Solution-Process Exciplex OLEDs

**DOI:** 10.3390/polym13101668

**Published:** 2021-05-20

**Authors:** Zong-Liang Tseng, Wei-Lun Huang, Tzu-Hung Yeh, You-Xun Xu, Chih-Hsun Chiang

**Affiliations:** 1 Department of Electronic Engineering, Ming Chi University of Technology, New Taipei City 243303, Taiwan; a88061446@gmail.com (W.-L.H.); sasa220404373@gmail.com (Y.-X.X.); u06157019@mail2.mcut.edu.tw (C.-H.C.); 2 Organic Electronics Research Center, Ming Chi University of Technology, New Taipei City 243303, Taiwan; jim824645@gmail.com

**Keywords:** exciplex, TADF, OLED, light-emitting diode, RISC

## Abstract

Organic light-emitting diodes (OLEDs) have developed rapidly in recent years. Thermally activated delayed fluorescent (TADF) molecules open a path to increase exciton collection efficiency from 25% to 100%, and the solution process provides an alternative technology to achieve lower cost OLEDs more easily. To develop commercial materials as exciplex hosts for high-performance and solution-processed OLEDs, we attempted to use 4,4′-cyclohexylidenebis[N,N-bis(4-methylphenyl)benzenamine (TAPC), poly(9-vinylcarbazole) (PVK), N,N′-Di(1-naphthyl)-N,N′-diphenyl-(1,1′-biphenyl)-4,4′-diamine (NPB), and poly(N,N’-bis-4-butylphenyl-N,N’-bisphenyl)benzidine (Poly-TPD) as the donors and 2,4,6-tris[3-(diphenylphosphinyl)phenyl]-1,3,5-triazine (POT2T) as the acceptor to obtain the TADF effect. All donors and the acceptor were purchased from chemical suppliers. Our work shows that excellent TADF properties and high-efficiency exciplex OLEDs with low turn-on voltage and high luminance can be achieved with a simple combination of commercial materials.

## 1. Introduction

Thermally activated delayed fluorescent (TADF) molecules [1,2,3,4] open a path to increasing exciton collection efficiency from 25% to 100%. Since the first report was presented in 2012 [1], TADF organic light-emitting diodes (OLEDs) have also made significant breakthroughs over the past years, such as external quantum efficiency (EQE) of the electroluminescence device over 30%. For example, the blue OLEDs exhibited an EQE of 37% [5], which use a spiroacridine-triazine hybrid molecular to yield a highly efficient TADF effect. The color purity, and reduced roll-off of the TADF OLED with a high EQE of 31% was achieved by designing and synthesizing a TADF molecule based on di(pyridin-2-yl)methanone cores as the electron-accepting units and di-tertbutyl-carbazole as the electron-donating units [6]. 5-(4-(4,6-diphenyl-1,3,5-triazin-2-yl)phenyl)-6,11,12-triphenyl-5,11-dihydroindolo [3,2-b] carbazole (TRZ-TPDICz) was synthesized and the TADF OLED using TRZ-TPDICz achieved a maximum EQE of 30.3% [7]. Most of the research efforts are concentrated on synthesizing new TADF molecules [8,9,10].

TADF characteristics were found in single molecules and an exciplex system [11]. The formation of an exciplex consists of individual electron-donating (D) and electron-accepting (A) molecules, in which the highest occupied molecular orbital (HOMO) is located on D molecules and the lowest unoccupied molecular orbital (LUMO) is located on A molecules [12,13]. Such exciplex systems require a proper energy level between the intermolecular charge-transfer (CT) state and the local triplet (LE) state [14,15] to minimize the loss of reverse intersystem crossing (RISC) for TADF properties. To date, it is still looking for which of the combinations of donors and acceptors can provide more effective RISC effect. Some of the research efforts kept designing and synthesizing new donor and acceptor molecules to find the better combination. However, many studies using commercially available materials, including donors and acceptors, to form exciplexes with the effective RISC property were reported [12,13,15,16,17,18,19]. On the other hand, all of the abovementioned studies used vacuum thermal evaporation (VTE) to form high-quality organic films for the higher device performance. However, because of the high material consumption and high cost in the VTE method, a low-cost solution-processed deposition is a feasible and effective strategy to prepare high-performance TADF OLEDs. Commercially available 2,4,6-tris[3-(diphenylphosphinyl)phenyl]-1,3,5-triazine (POT2T) is a potential candidate as a acceptor in the solution-processed exciplex system because of its high solubility, easy gain, and suitable LUMO level [20,21,22,23]. Table 1 collects the solution-processed exciplex OLEDs based on POT2T combined with different donor materials. Although the (4-(9-(perfluoropyridin-4-yl)-9*H*-fluoren-9-yl)-*N*,*N*-diphenylaniline (TPA-3):PO-T2T based OLED had achieved a high EQE of 14.4%, the TPA-3 is difficult to obtain because of the time-consuming and complicated synthesis process. Therefore, the feasible soluble exciplex combination of the commercial materials can provide a good reference for the future development of cost-effective exciplex OLEDs.

**Figure 1:**
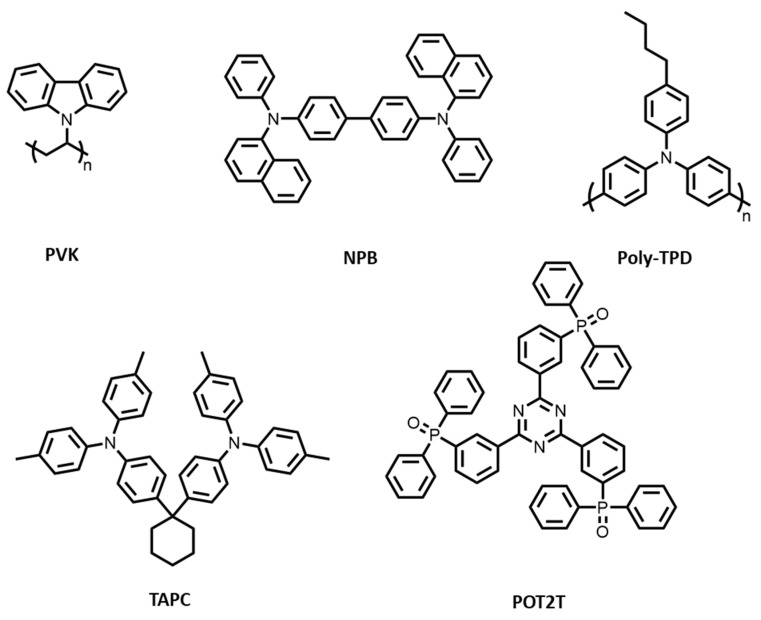
Chemical structures of donor and acceptor materials used in this work. 4,4′-cyclohexylidenebis[N,N-bis(4-methylphenyl)benzenamine (TAPC), poly(9-vinylcarbazole) (PVK), N,N′-Di(1-naphthyl)-N,N′-diphenyl-(1,1′-biphenyl)-4,4′-diamine (NPB), and poly(N,N’-bis-4-butylphenyl-N,N’-bisphenyl)benzidine (Poly-TPD) are used as the donors. 2,4,6-tris[3-(diphenylphosphinyl)phenyl]-1,3,5-triazine (POT2T) is used as the acceptor.

In this work, we attempted to use commercially available materials as the donors (TAPC, PVK, NPB, and Poly-TPD) and acceptor (POT2T) to form solution-processed exciplex systems (Figure 1). The properties of TADF and physical mechanisms in blended films and the corresponding solution-processed devices are discussed. The results show that excellent TADF properties and high-efficiency exciplex-OLEDs with low turn-on voltage and high luminance can be achieved with a simple combination of commercial materials.

## 2. Experimental Section

Poly(3,4-ethylenedioxythiophene) polystyrene sulfonate (PEDOT:PSS, AI 4083) and poly(9-vinylcarbazole) (PVK) were purchased from Sigma-Aldrich. 4,4′-Cyclohexylidenebis[N,N-bis(4-methylphenyl)benzenamine (TAPC) and N,N′-Di(1-naphthyl)-N,N′-diphenyl-(1,1′-biphenyl)-4,4′-diamine (NPB) were purchased from Shine Materials. Poly(N,N’-bis-4-butylphenyl-N,N’-bisphenyl)benzidine (Poly-TPD) and 2,4,6-tris[3-(diphenylphosphinyl)phenyl]-1,3,5-triazine (POT2T) were purchased from LUMTEC. All chemicals were used directly without further purification.

OLED devices were constructed with the architecture of indium tin oxide (ITO), PEDOT:PSS (40 nm), donor–acceptor blends (D:A = 2:1; 40 nm), POT2T (60 nm), LiF (1 nm), and Al (100 nm). The blends were TAPC:POT2T (Dev1), PVK:POT2T (Dev2), NPB:POT2T (Dev3), and Poly-TPD:POT2T (Dev4). The patterned ITO substrates (18 mm × 28 mm) were cleaned by deionized water, acetone, and isopropyl alcohol for 30 minutes each, and then treated by O_2_ plasma cleaning for 10 min to remove the residual organic matter and improve the surface work function. After the O_2_ plasma treatment, PEDOT:PSS, used as the hole injection layer (HIL), was spin-coated at 8000 rpm for 40 s on the substrate and annealed at 130 °C for 15 min, resulting in a 40-nm thick layer. The emitting layers (EMLs), prepared using donor–acceptor blends (D:A = 2:1), were dissolved in chlorobenzene at a concentration of 32 mg/mL and then deposited onto the HIL by spin-coating at 6000 rpm for 40 s. The structure of Dev5, Dev6, Dev7, and Dev8 was ITO, PEDOT:PSS (40 nm), TAPC:PO-T2T (2:1), POT2T (60 nm), LiF (1 nm), and Al (100 nm). The TAPC:PO-T2T (2:1) layers were deposited with spinning speed of 2000, 4000, 6000, and 8000 rpm for Dev5, Dev6, Dev7, and Dev8, respectively. POT2T and LiF were used as the electron-transporting and electron-injecting layers, respectively, via vacuum evaporation deposition. After the deposition of organic layers, an Al cathode was deposited using a shadow mask to define the device area of 2 mm × 2 mm. Conventional glass encapsulation was performed in an N_2_-filled glove box to humidity. A cleaned 0.7-mm thick glass (10 mm × 25 mm) was used to cover the active area and UV resin was dispensed on the edges of the glass cover and cured using a UV lamp to seal the OLEDs for protection from oxygen and humidity.

Electroluminescence (EL) spectra, current–voltage–luminance, and external quantum efficiency (EQE) were measured with a LQ-100R spectrometer (Enlitech) with computer control. Photoluminescence (PL) spectra were measured with a FluoroMax-4 fluorescence spectrometer (Horiba Jobin Yvon). All measurements were carried out at room temperature and the devices were encapsulated in a glove box.

## 3. Results and Discussion

**Figure 2:**
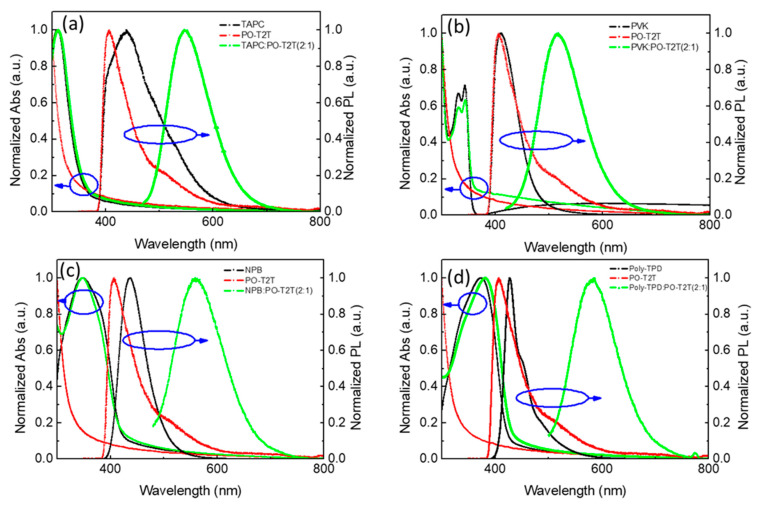
Absorption and photoluminescence (PL) spectra of donors, acceptor, and exciplexes obtained in solid films in air (for PL, λex = 330 nm): (**a**) TAPC:POT2T, (**b**) PVK:POT2T, (**c**) NPB:POT2T, and (**d**) Poly-TPD:POT2T.

**Figure 3:**
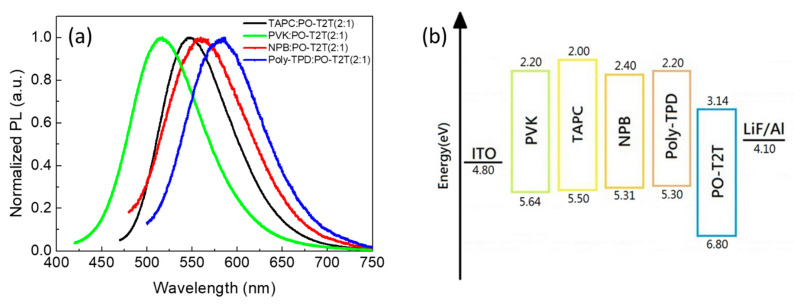
(**a**) Photoluminescence (PL) spectra of exciplex blended films; (**b**) energy levels of investigated donors and acceptors.

The chemical structure of the commercial materials studied in this work are shown in Figure 1. TAPC, PVK, NPB, and Poly-TPD were used as electron-donors and POT2T as electron acceptor, forming D:A combinations as exciplex hosts in this work. Figure 2 shows the photoluminescence (PL) characteristics of all materials, including donors, acceptor, and their blends. All donors and the acceptor showed blue emission, but red-shifted emission was observed in all the blends, indicating the presence of exciplexes in all D:A combinations. It is noted that no residue emissions from individual D and A components could be found in the blended films. This may be attributed to the fact that exciton emissions only from the CT states of exciplexes appeared, implying good exciplex formation with pure color through the combinations of commercial materials. Figure 3a shows the different wavelengths of emissions from the blended films. This is because the ionization potential (IP_D_) of the donor and the electron affinity (EA_A_) of the acceptor determine the energy of the CT state between exciplex molecules [26,27]. These are generally identified as the energy difference between the HOMO of the donor and the LUMO of the acceptor [28]. The difference in the emission wavelengths is attributed to the energy level IP_D_ − EA_A_ of all exciplex blended films. When the IP_D_ − EA_A_ energy level difference is larger, the exciplex emission tends to blue shift. The IP_D_ of TAPC, PVK, NPB, and Poly-TPD was 5.50, 5.64, 5.31, and 5.30 eV, respectively. The EA_A_ of POT2T was 3.14 eV (Figure 3b). The details of the IP_D_ − EA_A_ difference of all blended films are listed in Table 2.

**Figure 4:**
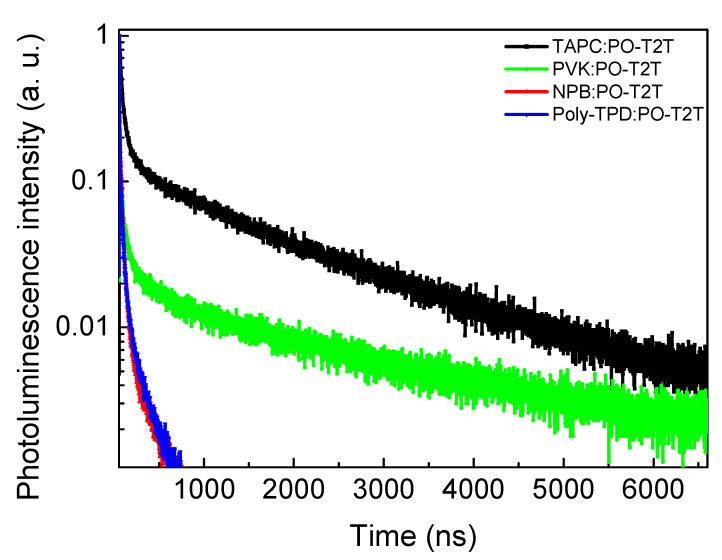
Room-temperature photoluminescence decay of investigated exciplex blends (λexc = 330 nm). All exciplexes are formed with a respective donor and POT2T as an acceptor. Donor:acceptor ratio is 2:1. Decays were recorded in atmosphere at 295 K.

As discussed above, the exciplex formation between the four commercial donor molecules and POT2T was found by PL spectroscopy measurement. To further verify the best combination of donor and POT2T molecules to form exciplex states, transient photoluminescence (TRPL) was used to confirm the exciton decay time of the blended films. The TRPL curves shown in Figure 4 are fitted by a three-exponential decay and average exciton lifetime (*τ*_av_), summarized in Table S1. The various donor molecules cause the obviously different variation tendency of the TRPL curves, implying different delayed fluorescence of exciplexes of the blended films. The *τ*_av_ of TAPC, PVK, NPB, and Poly-TPD/POT2T was 0.67, 0.61, 0.15, and 0.09 μs, respectively (see Table S1).

The *τ*_av_ of TAPC and PVK/POT2T was one order higher than that of NPB and Poly-TPD/ POT2T. Such behavior may be attributed to the significant difference of ΔE_ST_ in the various blends (Table 2). The small difference proves that the exciton in exciplex films could effectively transport from the triplet state to the singlet state, leading to obvious delayed fluorescence. TRPL identifies the exciton lifetime to confirm the TADF effect in the blended films. Therefore, a larger *τ*_av_ indicates higher reverse intersystem crossing (RISC) efficiency [26].

**Figure 5:**
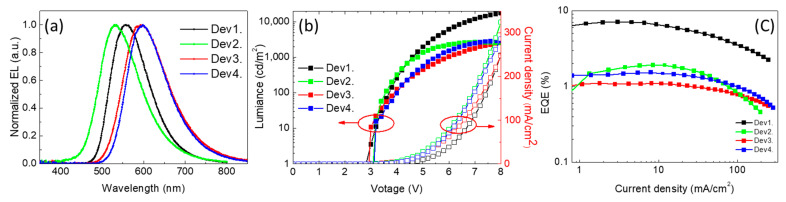
(**a**) Electroluminescence spectra, (**b**) external quantum efficiency (EQE) versus current density characteristics, and (**c**) current density–voltage–luminance curves of devices with different active layers. TAPC:PO-T2T, PVK:PO-T2T, NPB:PO-T2T, and Poly-TPB:PO-T2T devices are denoted as Dev1, Dev2, Dev3, and Dev4, respectively.

Figure 5a,b shows the normalized EL spectrum and current–voltage–luminance characteristics of the devices based on exciplex blended films as EMLs formed from commercial materials. The EL spectra of all devices are similar to the PL spectra of the corresponding blended films, showing the exciplex emissions. No significant difference in the EL peak profiles under different voltages (Figure S1) show the carriers strongly confined and recombined in the EMLs, indicating that stable devices could be prepared using commercial materials. The best EQE in Dev1 reaches 7.1%, as shown in Figure 5c, which is attributed to the low ΔE_ST_ in the TAPC:POT2T blend (Table 2). Compared to the device performances in the previous reports (Table 1), the excellent EQE of 7.1% in Dev1 demonstrates that the commercial TAPC:POT2T is a more feasible exciplex combination in solution process. This agrees with the long exciton lifetime in the TRPL results (Figure 4), which promotes the effective return of excitons in the triplet state to the singlet state, leading to efficient RISC. Although the PVK:POT2T blend exhibits the lowest ΔE_ST_ (Table 2), Dev2 shows an EQE of only 1.92%. This may be attributed to the small difference between the triplet state energy (2.96 eV) of the PVK [18] and the CT state (2.399 eV; Table 2) of the PVK:POT2T exciplex, leading to the exciton in the CT state going to the triplet state of the donor. The donors have higher triplet energy levels than the singlet energy of the exciplex state of blended films, leading to confinement of the CT state of the exciplex in the donor/POT2T blends. The large difference between the triplet state energy (2.87 eV) of the TAPC [29] and the CT state (2.272 eV; Table 2) of the TAPC:POT2T exciplex leads to the excellent performance of the TAPC/POT2T device. The detailed device performance is summarized in Table 3. Maximum EQE values of 1.1% and 1.51% were obtained for Dev3 and Dev4, respectively, due to large ΔE_ST_ in NPB:POT2T and Poly-TPD:POT2T and low exciton lifetime in the TRPL results (Figure 4). To further improve the device performance, the different thicknesses of the TAPC/POT2T layers were used to control the turn-on voltage (Von), EQE, and current density.

**Figure 6:**
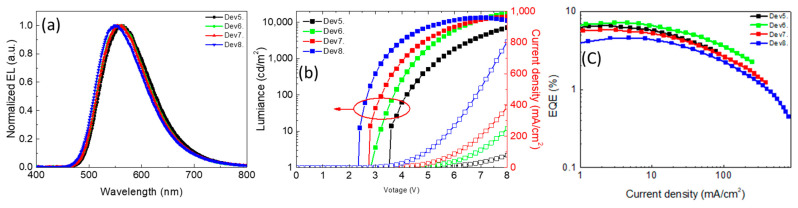
(**a**) Electroluminescence spectra, (**b**) EQE versus current density characteristics, and (**c**) current density–voltage–luminance curves of TAPC:PO-T2T devices with different thicknesses.

Figure 6 shows the lowest turn-on voltage (Von = 2.2 V) and highest current density in Dev8 due to the highest conductivity of the thinnest film. The slightly decreased EQE is seen in Dev5 with the largest thickness (Table 4), which may be caused by triplet–triplet annihilation (TTA) [30]. The highest EQE (7.1%) is obtained in Dev6, which is higher than the device prepared by the vacuum process in previous reports (Table 1) [24,25].

## 4. Conclusions

The use of commercial materials as exciplex hosts in solution-processed OLEDs has been demonstrated. This work presents a low-cost and simple method using commercial moleculars to achieve exciplex OLEDs. Obvious TADF behavior could be found in the combination of commercial moleculars such as TAPC/POT2T and PVK/POT2T blends. The highest EQE of 7.1% was achieved in the TAPC/POT2T devices because of the small ΔE_ST_ and long exciton lifetime as shown by TRPL.

## Figures and Tables

**Table 1:** Summary of recent reports of donor: PO-T2T based OLEDs.

Year	V_on_ (V)	EML	CE (cd/A)	PE (lm/W)	EQE (%)	Method	Reference
2018	4.9	PVK:PO-T2T	13.3	-	4.5	Solution process	[20]
2018	3	TAPC:PO-T2T	11.8	11.8	5.1	Vacuum deposition	[24]
2019	2.4	TPA-3:PO-T2T	44.8	41.5	14.4	Solution process	[21]
2019	2.3	DTF:PO-T2T	19.7	24.7	6	Solution process	[21]
2020	3.5	PVK:PO-T2T	15	7	4.75	Solution process	[22]
2020	3.5	mCP:PO-T2T	6.7	5.4	3	Solution process	[23]
2020	3	PVK:PO-T2T	14.8	9.3	4.6	Solution process	[23]
2020	3	TAPC:PO-T2T	4.49	4.21	1.67	Vacuum deposition	[25]
2021	2.8	TAPC:PO-T2T	17.2	16.9	7.1	Solution process	This work

**Table 2:** Photophysical Properties of Investigated Exciplexes.

Exciplex	IP_D_ − EA_A_, eV ^a^	CT, eV ^b^	LT, eV ^c^	ΔE_ST_, eV ^d^
TAPC:POT2T	2.36	2.272	2.248	0.024
PVK:POT2T	2.50	2.399	2.382	0.017
NPB:POT2T	2.17	2.215	2.112	0.103
Poly-TPD:POT2T	2.16	2.116	2.087	0.029

^a^ Ionization potential of donor (IP_D_)—electron affinity of acceptor (EA_A_) difference (energy difference between the highest occupied molecular orbital (HOMO) of the donor and the lowest unoccupied molecular orbital (LUMO) of the acceptor [19]). ^b^ Singlet state energy of blend = 1240/wavelength of PL peak at 77 K (established from Figure S1 in the Supplementary Material). ^c^ Triplet state energy of blend = 1240/wavelength of PL peak at 77 K (established from Figure S1). ^d^ Singlet−triplet energy level difference of blend (difference between singlet and triplet state energy of blend).

**Table 3:** Properties of organic light-emitting diode (OLED) devices for device structure: ITO, PEDOT:PSS (40 nm), exciplex emission (30 nm), POT2T (60 nm), LiF (1 nm), and Al (100 nm).

Device	EL (nm)	Turn-On Voltage, at 1 cd/m^2^	Maximum EQE/EQE at 10 mA/cm^2^ (%)	Maximum Power Efficiency/Power Efficiency at 10 mA/cm^2^ (lm/W)
Dev1	556	2.81	7.10/6.53	16.9/12.94
Dev2	535	2.82	1.92/1.91	4.43/4.16
Dev3	593	3.01	1.10/1.09	2.08/1.50
Dev4	598	3.01	1.53/1.49	3.56/2.01

**Table 4:** Properties of OLED devices for device structure: ITO, PEDOT:PSS (40 nm), TAPC:PO-T2T (2:1) with different thicknesses, POT2T (60 nm), LiF (1 nm), and Al (100 nm).

Device	Thickness (nm)	Turn-On Voltage, at 1 cd/m^2^	Maximum EQE/EQE at 10 mA/cm^2^ (%)	Maximum Power Efficiency/Power Efficiency at 10 mA/cm^2^ (lm/W)
Dev5	43 ± 2	3.41	6.48/5.68	13.51/8.92
Dev6	36 ± 2	2.81	7.10/6.53	16.90/12.94
Dev7	27 ± 3	2.62	5.75/5.22	16.06/11.49
Dev8	14 ± 2	2.22	4.61/4.37	15.10/12.52

## Data Availability

The data presented in this study are available on request from the corresponding author.

## Supplementary Materials

The following are available online at https://www.mdpi.com/article/10.3390/polym13101668/s1, Figure S1: Phosphorescence spectrum of (a) TAPC:POT2T (b) PVK:POT2T (c) NPB:POT2T (d) Poly-TPD:POT2T at 77K., Figure S2. Electroluminescence spectra of fabricated devices at various driving voltages; (a) TAPC:POT2T, (b) PVK:POT2T, (c) NPB:POT2T, (d) Poly-TPB:POT2T., Table S1. Average exciton lifetime of Investigated Exciplexes.
