# Supplementary material for: Thermally Activated Delayed Fluorescence in Commercially Available Materials for Solution-Process Exciplex OLEDs
Source: Polymers (Basel). 2021 May 20;13(10):1668. doi: 10.3390/polym13101668 (PMC8160893; doi:10.3390/polym13101668)
Supplement: Supplementary file 1 [file polymers-13-01668-s001.zip › polymers-1124356-supplementary.pdf]

Supporting Information

Thermally Activated Delayed Fluorescence in Commercially Available Materials for Solution-Process Exciplex OLEDs

Zong-Liang Tseng[1, 2,*], Wei-Lun Huang[1], You-Xun Xu[1], Chih-Hsun Chiang[1]

[1]Department of Electronic Engineering and [2]Organic Electronics Research Center, Ming Chi University of Technology, New Taipei City 24301, Taiwan (R.O.C)

Corresponding authors' e-mail:

zltseng@mail.mcut.edu.tw (Z. L. Tseng)

Table S1. Average exciton lifetime of Investigated Exciplexes

Exciplex	τ_{av} (s)
TAPC:POT2T	6.70×10^{-7}
PVK:POT2T	6.10×10^{-7}
NPB:POT2T	1.53×10^{-7}
Poly-TPD:POT2T	9.41×10^{-8}

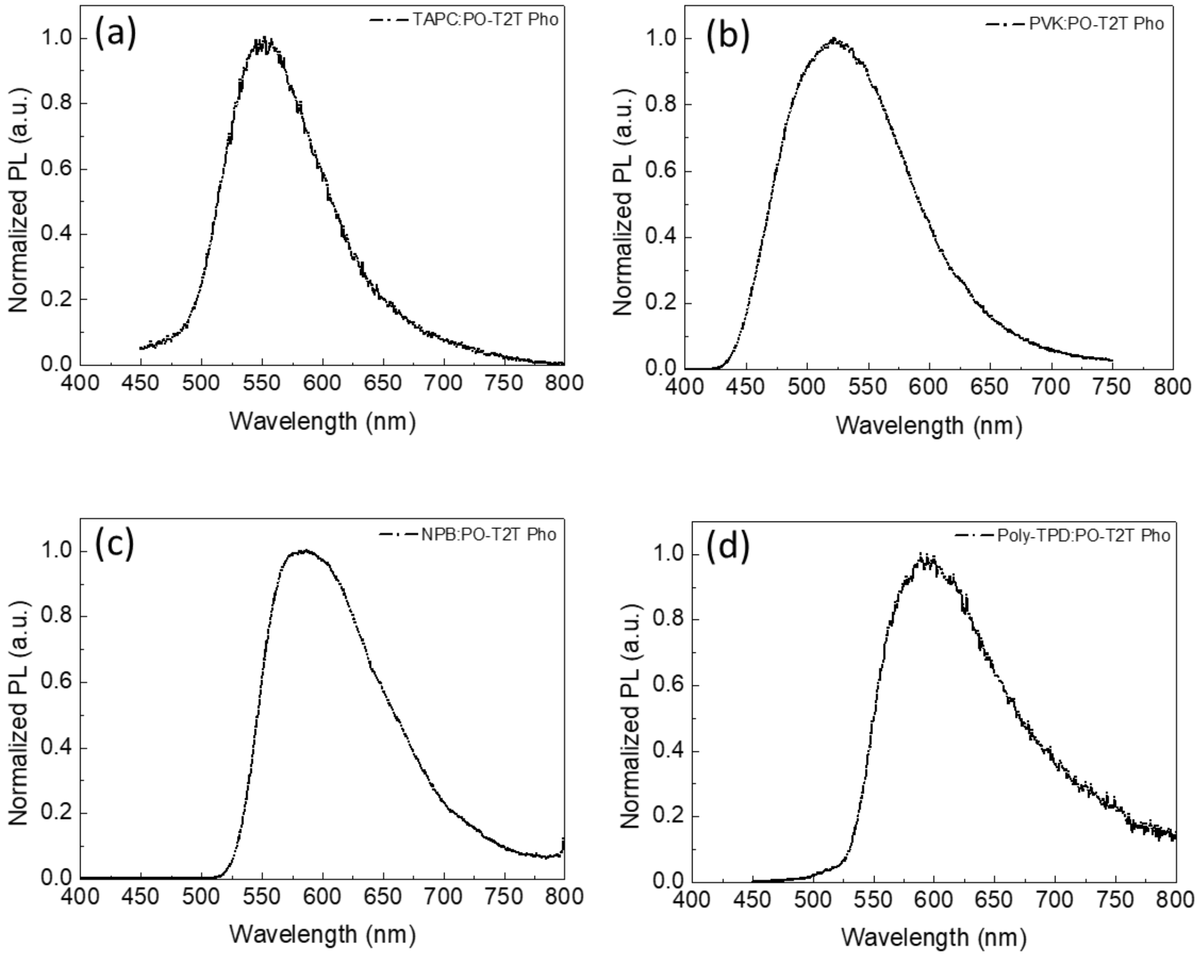

Figure S1. Phosphorescence spectrum of (a) TAPC:POT2T (b) PVK:POT2T (c) NPB:POT2T (d) Poly-TPD:POT2T at 77K.

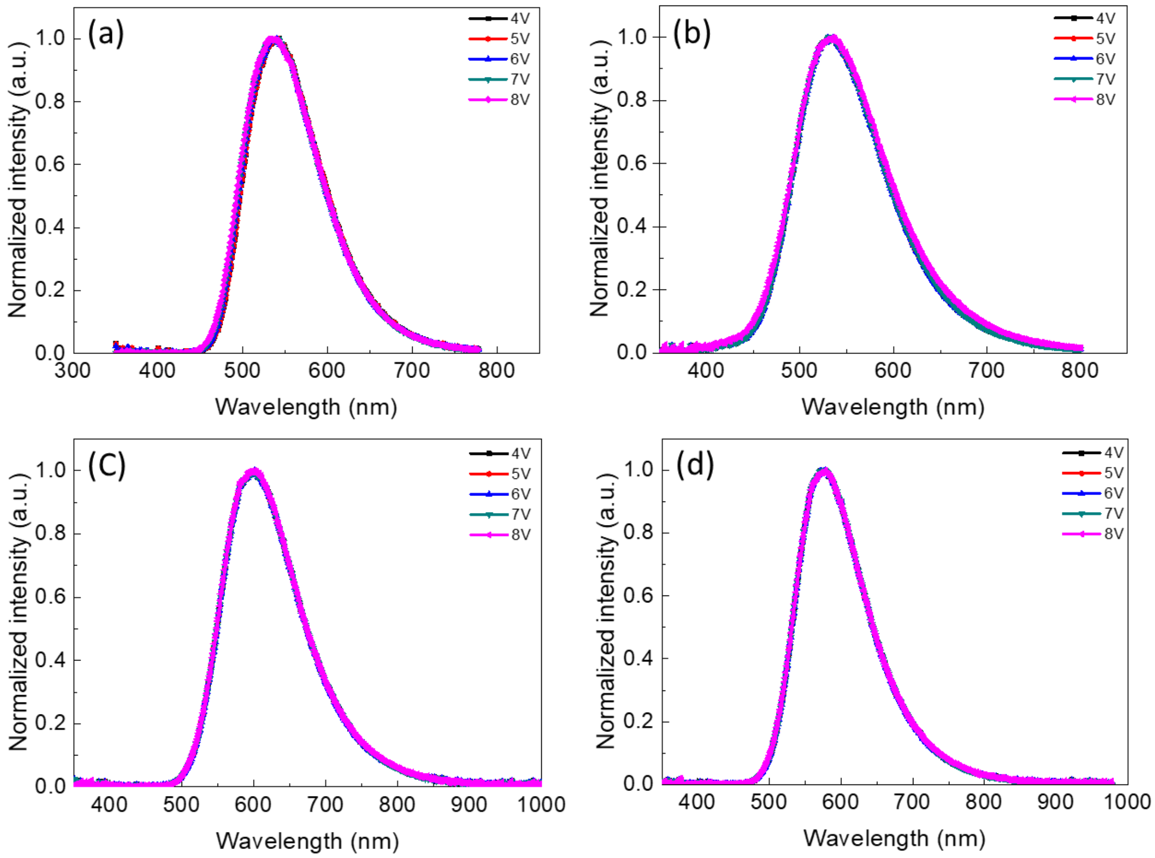

Figure S2. Electroluminescence spectra of fabricated devices at various driving voltages.; (a) TAPC:POT2T, (b) PVK :POT2T, (c) NPB:POT2T, (d) Poly-TPB:POT2T.
